# Safety and feasibility of outpatient autologous stem cell transplantation in pediatric patients with primary central nervous system tumors

**DOI:** 10.1038/s41409-019-0479-3

**Published:** 2019-02-19

**Authors:** Jane Koo, Stacy Silverman, Brandon Nuechterlein, Amy K. Keating, Michael R. Verneris, Nicholas K. Foreman, Jean M. Mulcahy Levy

**Affiliations:** 1 Department of Pediatric Hematology/Oncology/Bone Marrow Transplant, University of Colorado, Children’s Hospital Colorado, Aurora, CO USA; 2 The Morgan Adams Foundation Pediatric Brain Tumor Research Program, Children’s Hospital Colorado, Aurora, CO USA

**Keywords:** Haematopoietic stem cells, Cancer

## Abstract

High-dose chemotherapy with autologous hematopoietic stem cell transplantation (autoHSCT) is a well-established treatment for pediatric central nervous system (CNS) tumors. Given the risks of toxicity and infection, pediatric autoHSCT has been historically performed on hospitalized children. As our practice evolved, some patients were transplanted as outpatients. We performed a retrospective cohort analysis of 37 patients who received 90 transplant procedures (49 outpatient and 41 inpatient) at Children’s Hospital Colorado. The most common primary diagnosis was medulloblastoma (51.4%). Of the patients transplanted as outpatients, 69.4% were admitted for fever and neutropenia and had a median time to hospitalization of day +6, with fever and neutropenia being the most common reasons for admission. The median time to neutrophil engraftment was the same in both cohorts, 11 days. Median time to platelet engraftment was 13 days (8–82 days) vs 16 days (8–106 days) (*p* = 0.0008). At day +100, the transplant-related mortality (TRM) was 0% for both the cohorts. At a median follow-up of 1.7 years, overall survival (OS) for all patients was 66.1% and TRM was 0% for both the cohorts. Outpatient autoHSCT for properly selected children with CNS tumors is safe and effective.

## Introduction

High-dose chemotherapy with autologous hematopoietic stem cell transplantation (autoHSCT) is a well-established treatment for multiple types of pediatric central nervous system (CNS) tumors [1–13]. Bacterial infections continue to be a major cause of morbidity and mortality in pediatric patients undergoing high-dose chemotherapy and autoHSCT. The incidence of bacteremia prior to engraftment in all types of pediatric transplant recipients is considerable and still accounts for significant morbidity and mortality. Previously, the incidence of bacteremia, specifically in pediatric autoHSCTs, has been reported to range from 24 to 34% [14, 15]. More recently, in the era of antibiotic prophylaxis, the incidence of bacteremia for autoHSCT patients is reported to range from 7 to 15% [16–18]. Information on infectious complications in pediatric CNS tumor patients treated with autoHSCT is quite limited, with only one dedicated study analyzing infections in this population, published in 2013, showing that catheter-related gram-negative bacteremia was the most common infectious complication [19].

Previously, autoHSCT has been strictly performed in the inpatient setting given the concerns for transplant-associated toxicity and infection. The safety and feasibility of outpatient autoHSCT for pediatric CNS tumor patients have not been considered. In adults, outpatient autoHSCT is routinely used in patients with multiple myeloma and lymphoma and has resulted in excellent outcomes [20–26]. However, performing outpatient transplantation in young children presents unique challenges, including an inability to articulate their clinical status and their well-recognized clinical deterioration in the setting of sepsis relative to adolescents and adults. To date, no study has analyzed the outcomes and infectious complications of pediatric CNS tumor patients receiving autoHSCTs in the outpatient setting. Due to the lack of evidence in the literature, we retrospectively compared the safety and efficacy of autoHSCTs in the outpatient setting for pediatric CNS tumor patients.

## Methods

### Patients

We performed a retrospective cohort study in all the pediatric patients with primary CNS tumors who received an autologous stem cell transplant between January 2011 and July 2017 at the Center for Cancer and Blood Disorders (CCBD) at Children’s Hospital Colorado. All data were extracted from the electronic medical record. The decision to analyze patients starting from 2011 was due to the establishment of the electronic medical record allowing for the standardized evaluation of patient records. Transplant episodes were categorized as inpatient if the stem cell infusion occurred while admitted to the hospital and outpatient if both the conditioning regimen and stem cell infusion occurred in the outpatient infusion center. Patients provided written informed consent for the procedure. This study was approved by the Institutional Review Board at the University of Colorado (IRB 05-0149).

### AutoHSCT procedure in the outpatient setting

Patients undergoing autoHSCT in the outpatient setting received all the care in the outpatient clinic within Children’s Hospital Colorado. According to our internal algorithms, patients with stable medical conditions, a Karnofsky/Lansky Performance Status (KPS/LPS) score of > 60, were expected to have manageable gastrointestinal toxicities from the preparative regimen, the primary caregiver was assessed to be willing to provide outpatient therapy, and were within a reach of 60 min from the hospital. For patients who did not require admission to the hospital, outpatient follow-up visits occurred at least three times a week or more frequently as needed.

### Conditioning regimens

All patients received the standard chemotherapy regimen as delineated by the protocol determined by their primary diagnosis. The myeloablative chemotherapy dosing regimen for pediatric malignant brain tumors was carboplatin 17 mg/kg/day and thiotepa 10 mg/kg/day on days 1 and 2 with peripheral blood stem cell infusion on day 4 of each cycle or 48 h after the last thiotepa dose [27].

### Stem cell mobilization, collection, and infusion

Mobilization and collection of hematopoietic stem cells was performed based on established institutional protocols and guidelines. A minimum of 2 × 10^6^ CD34^+^/kg stem cells were administered for each autologous transplant with a maximum of 5 × 10^6^ CD34^+^ /kg stem cells.

### Infection prophylaxis and treatment

Patients undergoing inpatient autoHSCT received acyclovir and fluconazole for anti-viral and anti-fungal prophylaxis beginning from day +1. Acyclovir and fluconazole were discontinued at the time of discharge. The outpatient cohort did not receive acyclovir or fluconazole prophylaxis. In the inpatient setting, bacterial prophylaxis was intravenous (IV) meropenem or cefepime starting when the Absolute Neutrophil Count (ANC) was < 500/mm^3^ or with the development of fever. For *Pneumocystis jiroveci* pneumonia (PJP) prophylaxis, IV pentamidine was started on day +25 and administered every 3 weeks. Prophylactic pentamidine was discontinued when the absolute lymphocyte count was greater than 1000/mm^3^.

### Other supportive care

All the inpatients received granulocyte colony-stimulating factor (G-CSF) at 5 mcg/kg/dose every 24 h beginning on day +1 and continued daily until the ANC was greater than 2500/mm^3^ for 3 consecutive days. For outpatient transplants, all patients received pegfilgrastim (Neulasta) dosed appropriately for their weight on day +1. With regard to transfusions, packed red blood cells were administered for a hemoglobin count of less than 8.5 g/dL. Platelet transfusions were administered for a platelet count of less than 20,000/mm^3^ or symptomatic bleeding. Scheduled and as-needed antiemetics were administered for patients actively receiving chemotherapy and to help control symptoms of nausea and vomiting. IV fluids were administered with chemotherapy as outlined in the protocol.

### Study endpoints

Each transplant episode was treated as a separate encounter and classified as outpatient or inpatient. For each episode, the day from infusion on which the patient was admitted was documented. Duration of hospitalization, antibiotics initiated, the total number of days of antibiotic use, the number of blood cultures drawn, and the details of any infection were tabulated, including the site and type of infection, and the number of days from stem cell infusion was documented. The number of days spent in the ICU was also recorded.

### Engraftment

Neutrophil engraftment was defined as the first day of an ANC above 500/mm^3^ for 3 consecutive days. Platelet engraftment was similarly defined as a platelet count greater than 20,000/mm^3^ for 7 consecutive days without requiring a transfusion.

### Infection

An infectious disease event was defined as any bacterial, viral, or fungal infection that was confirmed by laboratory testing, radiographic imaging, or physical exam and that required antimicrobial treatment. A central line-associated blood stream infection (CLABSI) was defined according to the Center for Disease Control guidelines (recovery of a pathogen from a blood culture (a single blood culture for organisms not commonly present on the skin and two or more blood cultures for organisms commonly present on the skin) in a patient who had a central line at the time of infection or within a 48-h period before the development of infection). The infection could not be related to any other infection the patient might have and must not have been present or incubating when the patient was admitted to the facility [28].

### Statistical methods

Quantitative variables were reported as absolute numbers and percentages and expressed by the median and standard deviation, if normally distributed, and by the mean if not. Comparisons between groups were made using *t* test. Kaplan–Meier methods and log-rank tests were used to compare the overall survival, and neutrophil and platelet engraftment. Box and whisker plots and unpaired *t* test with Welch’s correction were created to compare the number of hospital and ICU days and the number of days of antibiotic use between the outpatient and inpatient cohorts. Mean and standard error of the mean were reported. All *p* values were two sided and considered statistically significant if < 0.05.

## Results

### Patient characteristics

A total of 37 pediatric patients who underwent autoHSCT for primary CNS tumors were analyzed. Patient and disease characteristics are shown in Table 1. The indications for transplantation for the entire cohort include medulloblastoma (54%), primitive neuroectodermal tumor (PNET, 19%), atypical teratoid/rhabdoid tumor (ATRT, 14%), non-germinomatous germ cell tumor (NGGCT, 8%), pineoblastoma (3%), and embryonal tumor with multilayered rosettes (ETMR, 3%). Among all the patients, the median age at diagnosis was 2 years (range 0.6–21 years) and the median age at the time of transplant was 3 years (range 1–23 years). Preparative regimens for patients are also shown in Table 1.

**Table 1:** Patient and disease characteristics

Characteristics	All patients
Number of patients, *n*	37
Gender, *n* (%)	
Male	22 (59.5)
Female	15 (40.5)
Ethnicity, *n* (%)	
Caucasian/white	29 (78.4)
Hispanic	5 (13.5)
Non-Hispanic native American	2 (5.4)
Mixed	1 (2.7)
Location of primary tumor, *n* (%)	
Posterior fossa region	21 (56.8)
Pineal region	8 (21.6)
Frontal lobe	1 (2.7)
Brainstem	2 (5.4)
Diffuse	3 (8.1)
Others	2 (5.4)
Primary diagnosis, *n* (%)	
Medulloblastoma	19 (51.4)
NGGCT	3 (8.1)
Pinealblastoma	1 (2.7)
PNET	8 (21.6)
ATRT	5 (13.5)
ETMR	1 (2.7)
Preparative regimens, *n* (%)	
Carboplatin/thiotepa	25 (67.6)
Vincristine/carboplatin/thiotepa	5 (13.5)
Others	7 (18.9)
Age at diagnosis, years, median (range)	2 (0.6–21)
Age at the time of autoHSCT, years, median (range)	3 (1–23)

Data presented are *n* (%), unless otherwise indicated

*HSCT* hematopoietic stem cell transplantation, *NGGCT* non-germinomatous germ cell tumor, *PNET* primitive neuroectodermal tumor, *ATRT* atypical teratoid/rhabdoid tumor, *ETMR* embryonal tumors with multilayered rosettes

### Characteristics of outpatient and inpatient transplant episodes

Differences in characteristics of outpatient and inpatient transplant episodes are shown in Table 2. In total, 49 transplant episodes occurred in the outpatient setting and 41 episodes occurred in the inpatient setting. The median age at transplant for the outpatient cohort was 2 years (range 1–10) and 4 years for the inpatient cohort (*p* = 0.005). The median KPS/LPS score for outpatient transplant episodes was 90 (range 70–100) compared to 80 (range 50–100) for inpatient transplant episodes (*p* = 0.007). Disease status at the time of transplant is also shown in Table 2. For the outpatient cohort, complete response was the most common disease status at the time of transplant (25 outpatient transplant episodes, 51%) compared to good clinical response as the most common disease status at the time of transplant for the inpatient cohort (17 inpatient transplant episodes, 41.5%) (*p* = 0.11). All patients transplanted in the outpatient setting were discharged from the clinic after stem cell infusion. Of the 41 inpatient transplant episodes, the most common reason for admission was family preference (53.7%) or physician preference (14.6%). The remaining reasons for the transition to inpatient status are detailed in Table 2. For transplant episodes in which physician preference for inpatient transplant was the documented reason, this decision was made solely on the capacity of the patients’ caregivers to perform outpatient care at home. More specifically, the decision to admit certain patients for transplant was taken if there was a history of a family being consistently unreliable with previous complex medical care prior to transplant. Other reasons for admission to the hospital for autoHSCT included monitoring for endocrine dysfunction and a decline in patient’s overall health by the third transplant.

**Table 2:** Outpatient and inpatient transplant characteristics

	Outpatient transplant episodes (*n* = 49)	Inpatient transplant episodes (*n* = 41)	*p* Value
Median age at transplant, years, median (range)	2 (1–10)	4 (1–23)	0.005
Karnofsky/Lansky Performance Status at the time of transplant, median (range)	90 (70–100)	80 (50–100)	0.007
Disease status at the time of transplant, *n* (%)			
CR	25 (51)	15 (36.6)	
NTR	4 (8.2)	1 (2.4)	0.11
GCR	0	17 (41.5)	
PR	13 (26.5)	3 (7.3)	
MD	1 (2)	1 (2.4)	
SD	1 (2)	1 (2.4)	
Relapse	2 (4.1)	2 (4.9)	
Refractory	1 (2)	1 (2.4)	
MR	2 (4.1)	0	
Reasons for inpatient autoHSCT, *n* (%)			
Family preference	-	22 (53.7)	
Physician preference		6 (14.6)	
Fever		5 (12.2)	
Complication from initial transplant		3 (7.3)	
Infection		1 (2.4)	
Renal insufficiency		2 (4.9)	
Others		2 (4.9)	

Data presented are *n* (%), unless otherwise indicated

*HSCT* hematopoietic stem cell transplantation, *CR* complete response, *NTR* near total response, *GCR* good clinical response, *PR* partial remission, *MD* minimal disease, *SD* stable disease, *MR* minimal response

### Hospital resource utilization

Utilization of hospital resources for all 90 transplant episodes is described in Table 3. Median days from transplant on admission for the outpatient cohort was day +6 (range + 2 to 33) and day -4 (range -8 to 20) for the inpatient cohort (*p* < 0.001). From the time of stem cell infusion until engraftment, a total of 34 (81%) outpatient transplant episodes required admission for febrile neutropenia, 3 (7.1%) for other vital sign instabilities, and 5 (11.9%) for other reasons. Other reasons included localized infection (cutaneous cellulitis and an infected urachus) and initiation of nasogastric tube feeds in the setting of significant weight loss and pain. The patients who were admitted to the hospital for other vital sign instabilities included those with hypotension and hypoxia without fever. Of the patients who received tandem transplants and had their initial autoHSCT in the outpatient setting, 19 (68.5%) of patients did not require admission on subsequent cycles. For the patients who required tandem transplants and had their initial autoHSCT in the inpatient setting, 5 (27.8%) of these patients had stem cell infusions performed in the outpatient setting on subsequent cycles.

**Table 3:** Hospital-resource utilization between outpatient and inpatient transplant episodes

	Outpatient transplant episodes (*n* = 49)	Inpatient transplant episodes (*n* = 41)	*p* Value
Days from transplant on admission, median (range)	+6 (+2 to 33)	−4 (−8 to 20)	< 0.001
Primary reason for hospital admission after outpatient autoHSCT, *n* (%)			
Planned admission for HSCT	0		
Febrile neutropenia	34 (81.0)		
Vital sign instability (no fever)	3 (7.1)		
Others	5 (11.9)		
Initial outpatient transplant followed by inpatient transplant, *n* (%)	6 (31.5)	-	
Initial inpatient transplant followed by outpatient transplant, *n* (%)	-	5 (27.8)	
Number of days in the hospital, median (range)	6 (0–21)	17 (2–141)	< 0.001
Transplant episodes with ICU admissions, *n* (%)	9 (18.4)	8 (19.5)	0.89
Day post-autoHSCT when admitted to ICU, median (range)	+6 (+3 to 33)	+11 (0–27)	0.75
Length of ICU stay, median (range)	0 (0–4)	0 (0–37)	0.13
Indication for ICU admission, *n* (%)			0.62
Vital sign abnormality	6 (66.7)	4 (50)	
Respiratory insufficiency/failure	2 (22.2)	3 (37.5)	
Altered mental status	1 (11.1)	1 (12.5)	

*HSCT* hematopoietic stem cell transplantation

Of all the patients admitted to the hospital, either for an inpatient transplant or upon admission following outpatient transplantation, nine and eight patients (18.4 vs 19.5%, *p* = 0.89) required ICU admission. Median time to ICU admission from autoHSCT was day +6 for the outpatient cohort and day +11 for the inpatient cohort (*p* = 0.75). The most common indication for ICU admission was vital sign abnormality for both the outpatient and inpatient cohorts (66.7 vs 50%, *p* = 0.62). Of the patients with vital sign abnormality, five patients in the outpatient cohort were admitted to the ICU for hypotension and one patient was admitted for persistent tachycardia in the setting of high fevers. Only one patient required 1 day of vasoactive medications to maintain adequate blood pressures. Within the outpatient cohort, two patients required transfer to the ICU for respiratory insufficiency/failure and one patient required 1 day of intubation. Within the inpatient cohort, four patients (50%) required transfer to the ICU for vital sign abnormality. Of these patients, three patients had hypotension and one patient had persistent tachycardia in the setting of high fevers. One of these patients required vasoactive medications for a total of 3 days to maintain adequate blood pressures. Three patients required transfer to the ICU for respiratory insufficiency/failure and two required mechanical ventilation. For both the patients who were transferred to the ICU for an altered mental status, one was thought to be secondary to septic shock and the other required manipulation of their external ventricular drain.

**Fig. 1:**
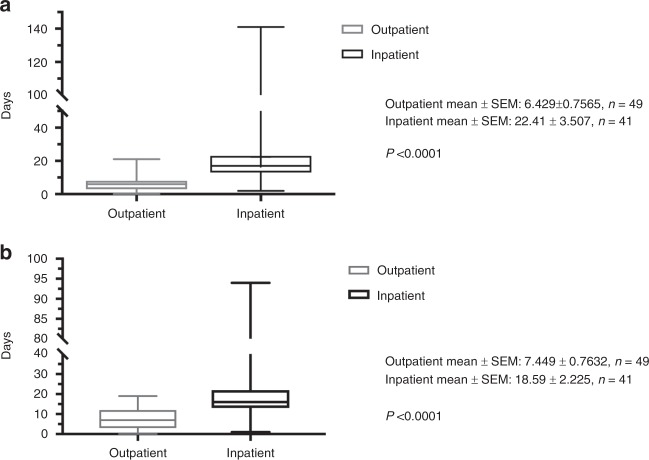
Box and whisker plots for **a** comparing the number of hospital days between outpatient and inpatient transplant episodes and **b** comparing the number of days of antibiotic use between outpatient and inpatient transplant episodes. SEM indicates standard error of the mean

The number of days spent in the hospital by the two cohorts is shown in Fig. 1a. The mean number of days spent in the hospital for the outpatient cohort was 6.4 days compared to 22.4 days for the inpatient cohort (*p* < 0.0001). The number of days of antibiotic use by the inpatient and outpatient cohorts is shown in Fig. 1b. The mean days of antibiotic use in the outpatient cohort was 7.4 days (range 0–19 days) and 18.6 days (range 1–94 days) in the inpatient cohort (*p* < 0.0001).

### Engraftment

**Fig. 2:**
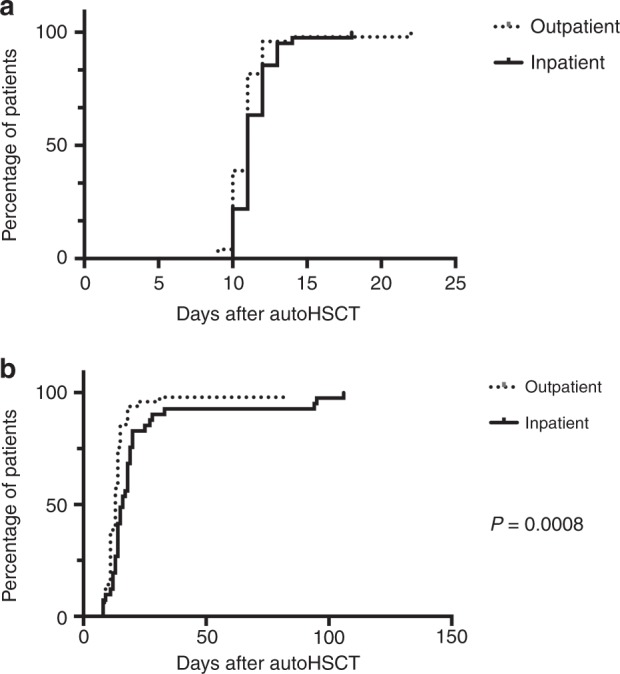
Kaplan–Meier curve for **a** neutrophil engraftment of outpatient and inpatient transplant episodes, and for **b** platelet engraftment of outpatient and inpatient transplant episodes

Early-transplant outcomes and characteristics, including neutrophil and platelet engraftment, are shown in Fig. 2a, b. Engraftment was achieved in 100% of all transplant episodes. Neutrophil recovery occurred at the same time for both the cohorts, with a median time to neutrophil recovery of 11 days. The median time to platelet recovery occurred earlier in the outpatient cohort (13 days vs 16 days, *p* = 0.0008).

### Infectious complications

Infectious complications, antibiotics initiated, duration of antibiotic therapy, the number of blood cultures drawn, and days of infectious complications for both transplant episodes are shown in Table 4. A total of 26 (53.1%) documented infections, including those from blood, stool, urine, and upper and lower respiratory tract, were isolated in the outpatient cohort, compared to 25 (67.6%) infections in the inpatient cohort. A majority of these were bacterial infections (80.8% outpatient vs 60% inpatient, *p* = 0.94). In the outpatient cohort, stool was the most common site of infection (46.2%), with *Clostridium difficile* being the most commonly isolated organism. For the inpatient cohort, blood was the most common site of infection (40%). Meropenem was the most common antibiotic initiated for both the cohorts (53.8% outpatient vs 87.5% inpatient, *p* = 0.01). For the outpatient cohort, the median time to isolate the infection was 6 days (range 2–28 days) compared to 7 days (range -5 to 120 days) for the inpatient cohort.

**Table 4:** Infectious complications of outpatient and inpatient transplant episodes

	Outpatient transplant episodes (*n* = 49)	Inpatient transplant episodes (*n* = 41)	*p* Value
Antibiotics initiated, *n* (%)			
Meropenem	21 (53.8)	35 (87.5)	
Cefepime	14 (35.9)	3 (7.5)	0.01
Ceftazidime	4 (10.3)	1 (2.5)	
Vancomycin	0	1 (2.5)	
Clindamycin	0	0	
Total days of all antibiotics, median (range)	7 (0–19)	16 (1–94)	< 0.0001
Sets of blood cultures drawn			
Mean ± SD	3.2 ± 3.48	5.15 ± 9.09	0.22
Number of total documented infections, *n* (%)	26 (53.1)	25 (67.6)	0.33
Sites of infection, *n* (%)			
CLABSI/blood	5 (19.2)	10 (40)	
Stool	12 (46.2)	7 (28)	
Skin	4 (15.4)	1 (4)	0.86
Urine/urologic	3 (11.5)	0	
Upper respiratory tract	2 (7.7%)	5 (20)	
Lungs	0	2 (8)	
Type of infection, *n* (%)			
Bacterial	21 (80.8)	15 (60)	
Viral	5 (19.2)	9 (36)	0.09
Fungal	0	1 (4)	
Days from transplant infection identified, median (range)	6 (2–28)	7 (−5–120)	0.08

*SD* standard deviation, *CLABSI* central line-associated bloodstream infection

### Survival

**Fig. 3:**
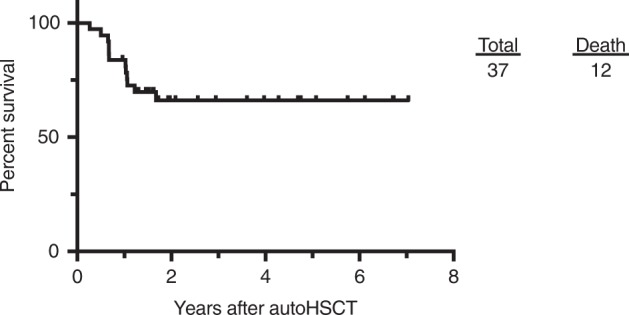
Kaplan–Meier curve for the overall survival of all the 37 patients

Figure 3 shows the OS for the entire cohort. At a median follow-up of 1.7 years (range 0.27–7.04 years) from the initial transplant for all patients, the OS was 66.1%. At a follow-up time of day +100, the transplant-related mortality (TRM) was 0% for both the cohorts. There were a total of 12 deaths. Eleven of these were from the progression of primary disease and one death was due to the complications related to radiation necrosis (Table 5).

**Table 5:** Cause of death

Cause	*n* (%)
Progressive disease, *n* (%)	11 (91.7)
Radiation necrosis, *n* (%)	1 (8.3)

Data presented are *n* (%) unless otherwise indicated

## Discussion

Our study of 37 patients shows the safety and efficacy of an outpatient transplant approach for autoHSCTs in medically stable pediatric CNS tumor patients. This is most clearly shown by the lack of TRM in both the outpatient and inpatient cohorts. Our results demonstrate that outpatient-based autoHSCT is feasible in selected cases and significantly reduces the length of hospital stay and antibiotic exposure, without an increase in complications.

This is the first analysis comparing the outcomes of pediatric CNS tumor patients undergoing autoHSCT in the outpatient and inpatient settings. There have been multiple reports in the adult literature describing a safe outpatient-based autoHSCT [29–33]. The experience with the outpatient management of autoHSCT in pediatric patients, more specifically CNS tumor patients, is not currently well described, and the data available are extrapolated from adult experience. The University of Montreal investigated the safety and efficacy of their outpatient program for autoHSCT in adult patients with multiple myeloma. All the 91 patients were treated on an identical outpatient protocol. Their results showed significant cost savings without a reduction in overall survival (100% at day +100 from transplant) [34]. Limited data from the pediatric setting has also attempted to demonstrate the feasibility of stem cell transplant in the ambulatory setting.

Previously, concerns for the safety of outpatient management of pediatric patients following high-dose conditioning regimens were included, but were not limited to mucositis, infections, and organ dysfunction, leading to the presumption that patients require a close monitoring within the hospital. Despite outpatient management, we observed a reduction in the number of serious infections. This is especially important as these patients did not receive an empiric antibiotic prophylaxis to prevent serious bacterial infections. We saw a reduced number of CLABSI in the outpatient cohort compared to that in the inpatient cohort. Possible considerations as to why this may have been observed in the outpatient cohort include decreased central-line manipulation, less-intensive monitoring of fever, perhaps leading to less-frequent initiation of antibiotics, and fewer virulent microorganisms that exist outside the hospital. The majority of outpatient transplant episodes (34/49, 81%) ultimately required readmission to the hospital for febrile neutropenia; however, none of these episodes resulted in death from infection. The discrepancy in the number of days of antibiotic use and the length of hospitalization stays between the outpatient and inpatient cohorts is largely explained by the initiation of antibiotics for any incidence of fever or ANC that dropped below 500/mm^3^ for all patients with transplants performed in the inpatient setting per our institutional guidelines. Additionally, the difference in the lengths of hospitalization stay between the outpatient and inpatient cohorts may also be explained by our institution’s practice of meeting the strict criteria of attaining an ANC > 2500/mm^3^ for 3 consecutive days before discontinuation of G-CSF in the inpatient setting. Also, the advantage of admitting patients within the outpatient cohort at the time of febrile neutropenia may have contributed to the decreased number of antibiotic days and hospitalization days.

The outpatient cohort also achieved a significantly faster platelet engraftment compared to the inpatient cohort. This difference may have been secondary to the prolonged use of G-CSF in the inpatient setting, which may have driven the development of hematopoietic precursor cells along the granulocyte pathway, potentially delaying the maturation of platelet progenitor cells. An additional reason may be that patients in the outpatient cohort were healthier at baseline and less heavily pre-treated prior to the time of transplant.

The lack of TRM and the low incidence of serious complications related to infection are likely due to the stringent compliance with our outpatient supportive-care practice. This is best demonstrated by the lower incidence of CLABSI in the outpatient cohort of all documented infections. However, the lack of TRM in the outpatient cohort also may reflect higher KPS/LPS scores and fewer co-morbidities, reflecting a healthier baseline status and a less-heavy treatment prior to transplant. Potential weaknesses of our study include the retrospective nature of this study, although patients were equally distributed based on age, diagnosis, and preparative regimen. Given the retrospective nature, patients were not randomized to have transplants in the outpatient or inpatient setting and were rather assessed by the treating physician, in conjunction with the family for the adequacy of outpatient transplant. It is possible that neither group identified the subtle characteristics not captured here, which led to the in- or outpatient treatment. Additionally, allowing parental preference to play a role in how transplants were performed (outpatient vs inpatient setting) adds a selection bias, favoring highly motivated caregivers wanting to limit the time within the hospital to receive outpatient therapy. Because our outpatient autoHSCT patients were carefully selected and monitored according to well-established and strict institutional outpatient-care guidelines, these results are not necessarily generalizable to all pediatric neuro-oncology patients.

This study demonstrates that outpatient autoHSCT is safe and efficacious for children with brain tumors. Such an approach can be applied to a vast majority of pediatric CNS tumor patients and has clinical outcomes comparable to those patients managed in the traditional inpatient setting. These data that we have presented here, however, are largely applicable to patients who have received carboplatin and thiotepa as a part of their conditioning regimen, which was used in a majority of our patients. Other myeloablative regimens may need a more intensive support that is not feasible in the outpatient setting. While beyond the scope of this analysis, it is clear that this approach has the potential to drive a significant reduction in the utilization of hospitalization resources.
